# Identification of the mitochondrial protein POLRMT as a potential therapeutic target of prostate cancer

**DOI:** 10.1038/s41419-023-06203-2

**Published:** 2023-10-10

**Authors:** Xiaojun Li, Linya Yao, Tao Wang, Xiaolei Gu, Yufan Wu, Ting Jiang

**Affiliations:** 1 Department of Urology, Taicang Affiliated Hospital of Soochow University, The First People’s Hospital of Taicang, Taicang, China; 2 Department of Urology, Kunshan Hospital of Traditional Chinese Medicine Affiliated to Yangzhou University, Kunshan, China

**Keywords:** Prostate cancer, Targeted therapies

## Abstract

RNA polymerase mitochondria (POLRMT) is essential for mitochondrial transcription machinery and other mitochondrial functions. Its expression and potential functions in prostate cancer were explored here. The Cancer Genome Atlas prostate cancer cohort (TCGA PRAD) shows that *POLRMT* mRNA expression is upregulated in prostate cancer tissues and *POLRMT* upregulation is correlated with poor patients’ survival. *POLRMT* mRNA and protein levels were upregulated in local prostate cancer tissues and different primary/immortalized prostate cancer cells. Genetic depletion of *POLRMT*, using viral shRNA or CRISPR/Cas9 gene editing methods, impaired mitochondrial functions in prostate cancer cells, leading to mitochondrial depolarization, oxidative stress, mitochondria complex I inhibition, and ATP depletion. Moreover, POLRMT depletion resulted in robust inhibition of prostate cancer cell viability, proliferation, and migration, and provoked apoptosis. Conversely, prostate cancer cell proliferation, migration, and ATP contents were strengthened following ectopic POLRMT overexpression. In vivo, intratumoral injection of POLRMT shRNA adeno-associated virus impeded prostate cancer xenograft growth in nude mice. POLRMT silencing, oxidative stress, and ATP depletion were detected in POLRMT shRNA-treated prostate cancer xenograft tissues. IMT1 (inhibitor of mitochondrial transcription 1), the first-in-class POLRMT inhibitor, inhibited prostate cancer cell growth in vitro and in vivo. Together, overexpressed POLRMT is an important mitochondrial protein for prostate cancer cell growth, representing a novel and promising diagnostic and therapeutic oncotarget.

## Introduction

Prostate cancer is still the second most common malignancy among men globally and is the fifth leading cause of cancer-related death [1, 2]. Prostate cancer’s incidence, progression, and mortality profiles vary significantly in people of different races and ethnicities [3, 4]. In the United States, prostate cancer ranks second in the mortality rate [1, 5]. In China and other developing countries, the incidence of this disease is rising [3, 4] and the firstly diagnosed cancers are already in the middle/late stages [3, 4].

The current clinical treatment options for prostate cancer include prostatectomy surgery, androgen deprivation therapy, and radiotherapy. These therapies can only temporarily limit the advance of prostate cancers [3, 4]. Within 18–24 months, cancers can progress to the metastatic stage [3, 4]. These patients often have poor prognosis with the average survival close to 12 months [3, 4]. The molecular pathology of this disease is very complicated and often involves dysregulation of multiple genetic, epigenetic, and proteomic factors [6–9]. It is therefore vital to further explore novel molecular/signaling targets for the oncogenesis and progression of prostate cancer [6–9].

Mitochondria are vital organelles for energy production, apoptosis regulation, and various other key cellular functions in prostate cancer [10]. Dysregulation of mitochondria is closely associated with increased incidence, therapy resistance, metastasis, and recurrence of prostate cancer [10]. The mitochondrial proteins are promising therapeutic targets of prostate cancer [10–12]. RNA polymerase mitochondria (POLRMT) is a mitochondrial protein essential for the mitochondrial transcription machinery [13, 14]. POLRMT assembles with two other components, mitochondrial transcription factor A (TFAM) and B2 (TFB2M), to initiate mitochondrial DNA (mtDNA) transcription [15, 16]. Furthermore, POLRMT participates in promoting RNA primer synthesis, required for the replication of mtDNA [14, 17]. It is also key in regulating oxidative phosphorylation (OXPHOS), mitochondrial biogenesis, and energy generation [15, 16].

Recent studies have proposed an essential role of POLRMT in the growth of different cancer cells. Bralha et al. showed that targeted inhibition or silencing of POLRMT impeded mitochondrial transcription, decreased mitochondrial gene expression and OXPHOS, and caused death in acute myeloid leukemia cells [18]. Han et al., have shown that POLRMT expression is elevated in human osteosarcoma tissues and cells, required for osteosarcoma cell growth. Contrarily, shRNA-mediated silencing or CRISPR/Cas9-induced knockout (KO) of POLRMT potently suppressed osteosarcoma cell growth in vitro and in vivo [19].

In addition, Zhou et al. discovered POLRMT overexpression in non-small cell lung cancer (NSCLC) tissues and cells. POLRMT shRNA or KO decreased cell viability, proliferation, and migration, and provoked apoptosis in primary and immortalized human NSCLC cells [20]. Wang et al. demonstrated that increased POLRMT expression in skin squamous cell carcinoma is required for maintaining mitochondrial functions and cancer cell growth [21]. Li et al., recently showed that IMT1(inhibitor of mitochondrial transcription 1), a first-in-class POLRMT inhibitor, robustly inhibited endometrial carcinoma cell growth in vitro and in vivo [22]. Nevertheless, the expression and potential functions of the mitochondrial protein POLRMT in prostate cancer have not been extensively studied thus far.

## Materials and methods

### Reagents

Polybrene, CCK-8, trypan blue, puromycin, medium, fetal bone serum, TRIzol, and antibiotics were provided by Sigma-Aldrich (St. Louis, MO). The cell fractionation antibody sampler kit was from Cell Signaling Tech (#11843, Shanghai, China). All other antibodies were provided by Dr. Wang [21]. IMT1 and fluorescence probes, including EdU (5-ethynyl-20-deoxyuridine), TUNEL (TdT-mediated dUTP Nick-End Labeling), JC*-*1 (tetraethylbenzimidazolylcarbocyanine iodide), DAPI and CellROX were provided by Dr. Li [22]. The BODIPY fluorescence probe was obtained from Invitrogen (Shanghai, China).

### Human tissues

The castration-resistant prostate cancer (CRPC) tissues and matched adjacent normal prostate epithelial tissues from ten written-informed consent primary patients were provided by Dr. Mi [23]. The protocols for testing human tissues were approved by the Ethics Committee of the First People’s Hospital of Taicang and were in according to the Declaration of Helsinki.

### Cells

The primary human prostate cancer cells, “pCan1” and “pCan2”, deriving from two written-informed consent CRPC patients, as well as the primary human prostate epithelial cells (“pEpi”) were provided by Dr. Mi [23]. Cells were cultivated using the described protocols [24]. The immortalized prostate cancer cell lines, LNCaP and PC-3, were provided by Dr. Tao [25]. The protocols for using primary human cells were approved by the Ethics Board of the First People’s Hospital of Taicang.

### Quantitative real-time PCR (qRT-PCR)

Brief, total cellular/tissue RNA was extracted by TRIzol reagents. RNA (1.0 μg per treatment) was reversely transcripted under a two-step RT-PCR kit (Takara Bio, Japan) [26]. Quantitative real-time PCR (qRT-PCR) was carried out by the 7900 PCR system (Applied Biosystems, Shanghai, China) under the SYBR GREEN PCR Master Mix (Thermo Fisher Scientific). *GAPDH* mRNA expression was always examined as the internal control. Data quantification was described previously [20]. Primers were from Dr. Zhou [20].

### Western blotting

Western blotting protocols were described elsewhere [27]. Each lane in SDS-PAGE gel was loaded with exact same amount of protein lysates (20 μg of each sample). The mitochondrial proteins were extracted via the mitochondria extraction kit (Sigma, Shanghai, China) by high-speed centrifugation. The uncropped blotting images are listed in Fig. S1.

### CCK-8 viability and Trypan blue cell death assays

Prostate cancer cells or epithelial cells with the described genetic treatments were first placed into 96-well plates with 4000 cells in each well. Thereafter, cells were maintained under 37 °C, 5% CO_2_ incubator for an additional 72 h. CCK-8 mixture (10 μL of each well) was then added. After another 2 h, the absorbance of CCK-8 was examined at 450 nm using a microplate reader. For quantifying cell death, the prostate cancer cells or epithelial cells were stained with Trypan blue. Trypan blue-stained “dead” cells were measured through an automatic cell counter.

### Caspase activity assay

Cells with the described genetic treatments were first placed into 6-well plates at 140,000 cells in each well. Thereafter, cells were cultured for an additional 72 h and cell lysates were obtained. The caspase-3 activity and the caspase-9 activity in the lysates (25 μg per treatment) were measured through Caspase-3/-9 Colorimetric Assay kits (BioVision, Milpitas, CA) based on the manufacturer’s instructions.

### “Transwell” assays

Prostate cancer cells with the described genetic modifications were re-suspended at 1.5 × 10^4^ cells per well in serum-free medium. Cells were thereafter placed on the upper surface of the “Transwell” chamber. The lower surface of the chamber was filled with 12% FBS-containing complete medium and cells were allowed to migrate for 24h. Afterwards, cells on the upper surface of the chamber were carefully wiped out with cotton swabs. Migrated cells on the lower surface were washed, fixed, and stained.

### Fluorescence staining

Cells with the described genetic treatments were first placed into the glass slide (in six well-plates) and were cultured for indicated time periods. The slides were then fixed with 4% paraformaldehyde and washed with PBS. Next, 0.5% Triton X-100 was included for permeabilization. Thereafter, different fluoresce dyes were added for 2 h and the slides were then washed with cold PBS. The fluoresce signalings were obtained using a fluorescence microscope (Zeiss), and their value was quantified.

### POLRMT silencing

Two different lentivirus-packed POLRMT shRNA, “shPOLRMT-S1” or “shPOLRMT-S2”, were provided by Dr. Zhou [20]. The virus was added (at MOI = 10) to cultured cells. Cells were then switched back to the fresh complete medium and puromycin was added to select stable cell colonies for 3–4 passages. POLRMT silencing in the stable cells was always verified. Control cells were infected with lentivirus encoding the scramble control shRNA sequence (“shC”, also provided by Dr. Zhou [20]).

### POLRMT knockout (KO)

Prostate cancer cells were cultivated in polybrene-containing complete medium and were infected with dCas9-expressing lentivirus (from Dr. Li [22]). Stable cells were established after selection using puromycin. Afterward, a lentiviral CRISPR/Cas9-POLRMT-KO construct (from Dr. Li [22]) was further transduced to the dCas9-expressing cells. Cells were then distributed into a 96-well plate and POLRMT KO was screened. Lastly, the POLRMT KO single stable cells were formed. Control cells were transduced with CRISPR/Cas9 control construct (“koC”, from Dr. Li [22]).

### PLORMT overexpression

To induce ectopic overexpression of POLRMT, prostate cancer cells were cultivated in polybrene-containing complete medium. The lentivirus encoding the POLRMT-overexpression construct (“oePOLRMT”), provided by Dr. Zhou [20], was added to the prostate cancer cells. Two days after virus treatment, cells were back to the fresh complete medium, and puromycin was then added to select stable cell colonies (for 3–4 passages). Two stable colonies were formed: “oePOLRMT-sSlc1” and “oePOLRMT-sSlc2”. Overexpression of PLORMT was verified by qRT-PCR and Western blotting assays. Control cells were infected with lentivirus-packed empty vector.

### Animal xenograft studies

All male nude mice (at 18.2–18.6 g, 5–6 weeks old) were purchased from Shanghai Laboratory Animal Center (SLAC, Shanghai, China). Six million prostate cancer cells per mouse were subcutaneously (s.c.) injected into nude mice’ flanks. After three weeks, the subcutaneous prostate xenografts were formed with each xenograft ~100 mm^3^ (labeled as “Day-0”). The xenograft-bearing nude mice were then intratumorally injected with POLRMT shRNA-expressing AAV (“aav-shPOLRMT-S1”, from Dr. Zhou [20]) or scramble control shRNA AAV (aav-c-sh) (from Dr. Zhou [20]). AAV injection was performed twice (at “Day-0” and “Day-2”). The estimated xenograft volumes, animal body weights, and estimated daily tumor growth (in mm^3^ per day) were calculated as described [20]. The superoxide dismutase (SOD) activity in the xenograft tissue lysates was analyzed by a commercial kit (Sigma) based on the attached protocol. Lipid peroxidation intensity in the prostate cancer tissue lysates was measured by a thiobarbituric acid reactive substance (TBAR) kit (Cayman Chemical, MI) [21]. All animal studies were approved by the IACUC and the Institute Animal Ethics Review Board of the First People’s Hospital of Taicang.

### Tissue immunofluorescence assays

For tissue immunofluorescence studies, the prostate cancer xenograft slides were blocked, washed, permeabilized, and stained with TUNEL; The slides were then counterstained with DAPI, washed, and visualized under the confocal microscope.

### Statistical analyses

Data in the present study were normally distributed and were always expressed as means ± standard deviation (SD). When comparing three or more groups, the one-way ANOVA and Scheffe’s *f*-test (SPSS 23.0) were utilized. For the two groups’ comparison, the two-tailed unpaired *t* test (Excel 2007) was utilized. *P* values < 0.05 were considered statistically significant.

## Results

### POLRMT upregulation in prostate cancer correlates with poor survival

**Fig. 1:**
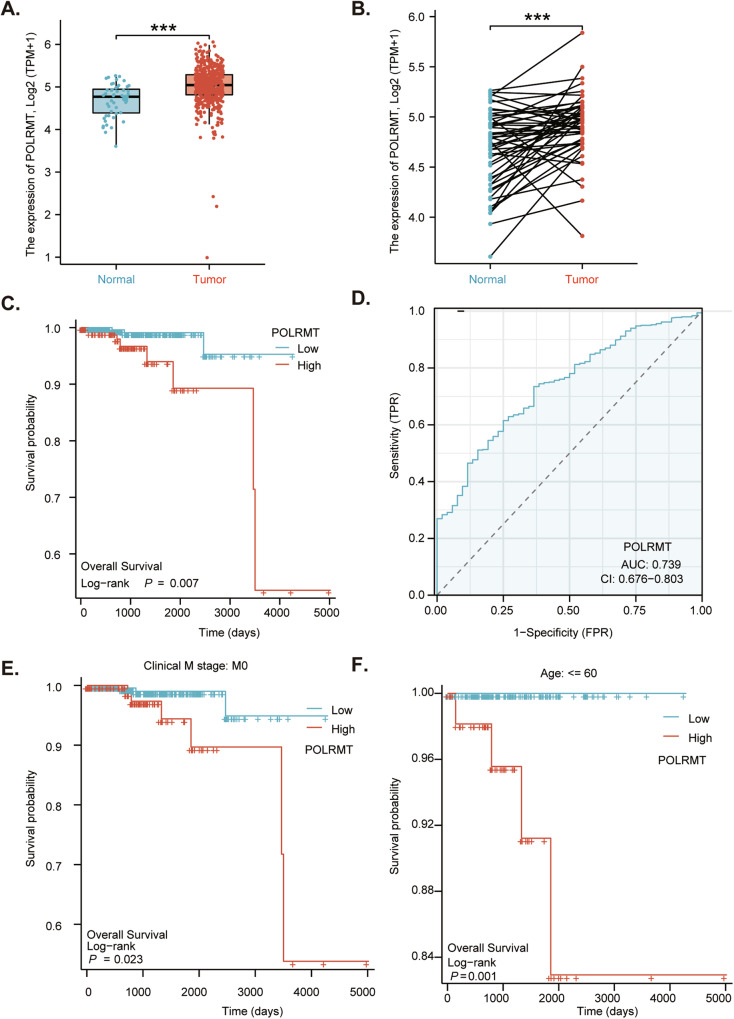
POLRMT upregulation in prostate cancer correlates with poor survival. The Cancer Genome Atlas (TCGA) prostate cancer cohort (TCGA PRAD) shows *POLRMT* expression (RNA-Seq) in prostate cancer tissues (“Tumor”, *n* = 501) and normal prostate tissues (“Normal”, *n* = 52) (**A**). TCGA PRAD shows *POLRMT* expression in prostate cancer tissues (“Tumor”, *n* = 52) and matched adjacent normal prostate tissues (“Normal”, *n* = 52) (**B**). The Kaplan-Meier survival analyses show the association between *POLRMT* expression and the overall survival of total prostate cancer patients (**C**) and subgroup prostate cancer patients (**E**, **F**). The receiver operating characteristic (ROC) curve results demonstrated the relationship between *POLRMT* overexpression and the potential predictive effect on prostate cancer patients’ survival (**D**).

Analyzing The Cancer Genome Atlas (TCGA) prostate cancer cohort (TCGA PRAD) revealed that the number of *POLRMT* transcripts in prostate cancer tissues (“Tumor”, *n* = 501) was significantly higher than that in the normal prostate tissues (“Normal”, *n* = 52) (Fig. 1A). Further TCGA PRAD analyses showed that expression of *POLRMT* in prostate cancer tissues (*n* = 52) was significantly higher than that in the matched adjacent normal tissues (*n* = 52) (Fig. 1B). Next, the Kaplan-Meier survival analysis was carried out and results demonstrated that high *POLRMT* expression in prostate cancer was correlated with poor overall survival (*P* = 0.008) (Fig. 1C). The receiver operating characteristic (ROC) curve analysis is an effective way to summarize the overall diagnostic accuracy of a potential oncogenic gene in human cancer. High *POLRMT* expression in prostate cancer tissues predicted poorer overall (area under curve/AUC = 0.739, Fig. 1D). The subgroup Kaplan–Meier survival analyses found that high *POLRMT* expression correlated with poor overall survival in patients with M = 0 cancer (Fig. 1E) or Age <= 60 (Fig. 1F). Thus, the studies indicate that *POLRMT* upregulation in prostate cancer correlates with poor survival.

### POLRMT upregulation in local prostate cancer tissues and in primary/immortalized prostate cancer cells

**Fig. 2:**
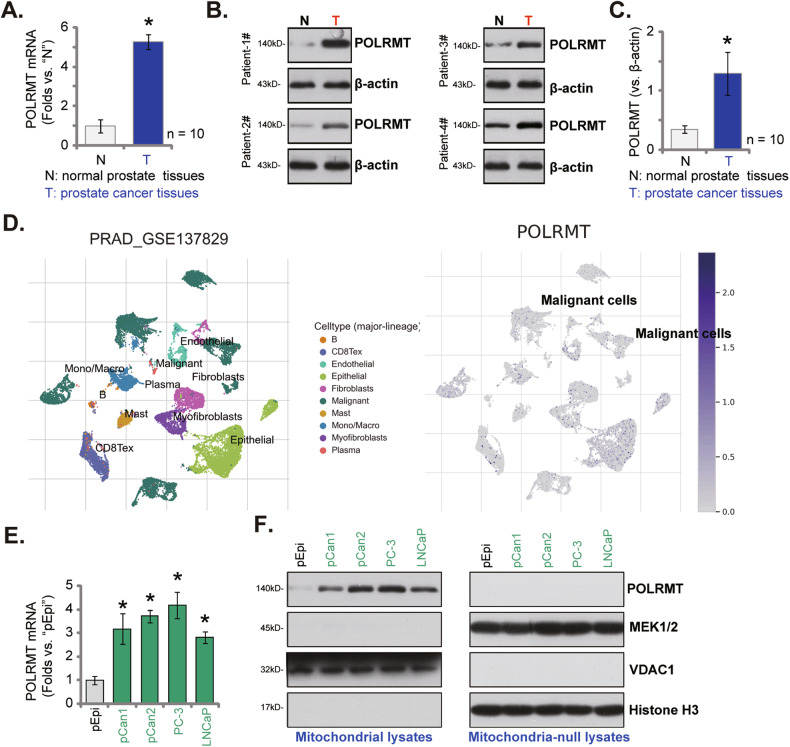
POLRMT upregulation in local prostate cancer tissues and primary/immortalized prostate cancer cells. *POLRMT* mRNA and protein expression in the described prostate cancer tissues (“T”) and matched adjacent normal prostate tissues (“N”) of ten (*n* = 10) primary patients was shown (**A**–**C**); The prostate cancer tumor microenvironment at single-cell resolution was characterized from GSE137829 database using the tumor immune single-cell hub2 (TISCH2) pipeline and relative expression of *POLRMT* in annotated cell types was shown (**D**). Expression of *POLRMT* mRNA (in total cell lysates) and protein (in both mitochondrial fraction lysates and mitochondria-null lysates) in the described prostate cancer cells and primary human prostate epithelial cells (“pEpi”) was shown (**E**, **F**). Error bars stand for mean ± standard deviation (SD). *P < 0.05 versus “N” tissues or “pEpi” cells. The experiments were repeated five times with similar results obtained.

The expression levels of POLRMT in local prostate cancer tissues were explored as well. The prostate cancer tissues (“T”) and matched adjacent normal tissues (“N”) (provided by Dr. Mi [23]) from a set of ten (10) written-informed primary prostate cancer patients were tested. In the prostate cancer tissues, *POLRMT* mRNA expression was significantly higher than that in the normal tissues (Fig. 2A). The protein expression of POLRMT in the fresh tissue lysates was examined by Western blotting assays and results confirmed POLRMT protein upregulation in prostate cancer tissues of four representative patients (“Patient-1” to “Patient-4”) (Fig. 2B). All blotting data of the ten sets of human tissues were combined and jointly analyzed. POLRMT protein upregulation in the cancer tissues was significant (*P* < 0.05 vs. “N” tissues) (Fig. 2C). These results are in line with the bioinformatical studies and supported POLRMT upregulation in prostate cancer.

We next used the tumor immune single-cell hub2 (TISCH2) pipeline to characterize prostate cancer tumor microenvironment at single-cell resolution (http://tisch.comp-genomics.org/) [28]. The GSE137829 database was utilized to annotate cell types, searching data from the original study [29]. The marker-based annotation method was employed in MAESTRO using the differentially expressed genes (DEGs) between clusters (InferCNV method) [28, 29]. Ten different cell clusters were annotated, including fibroblasts, malignant (cancer) cells, endothelial cells, exhausted CD8 T cells (CD8Tex), plasma cells, B cells, monocytes or macrophages (Mono/Macro), and others (Fig. 2D). As shown, relative expression of POLRMT in the malignant (cancer) cells is high among the cell clusters (Fig. 2D).

Next, the expression of POLRMT in different prostate cancer cells was examined as well. In patient-derived prostate cancer cells (“pCan1” and “pCan2”, from Dr. Mi [23]) and immortalized cell lines (PC-3/LNCaP), *POLRMT* mRNA expression is significantly higher than that in primary human prostate epithelial cells (“pEpi”, from Dr. Mi [23]) (Fig. 2E). Considering the POLRMT is a mitochondrial protein, mitochondrial fraction lysates of the prostate cancer cells/epithelial cells were separated. POLRMT protein was present in the mitochondrial lysates, where voltage-dependent anion channel 1 (VDAC1) was present (Fig. 2F), but MEK1/2 (the cytosol marker protein) and Histone H3 (the nuclear marker protein) were not (Fig. 2F). Importantly, the mitochondrial protein POLRMT was upregulated in primary/immortalized prostate cancer cells (Fig. 2G) and its expression is low in pEpi cells (Fig. 2F). POLRMT protein was not present in mitochondria-null lysates, indicated by the presence of MEK1/2 and Histone H3 (Fig. 2F), but absence of VDAC1 (Fig. 2F). These results supported mitochondrial POLRMT upregulation in human prostate cancer cells.

### POLRMT co-expressing genes (CEGs) and enriched pathways in TCGA prostate cancer database

**Fig. 3:**
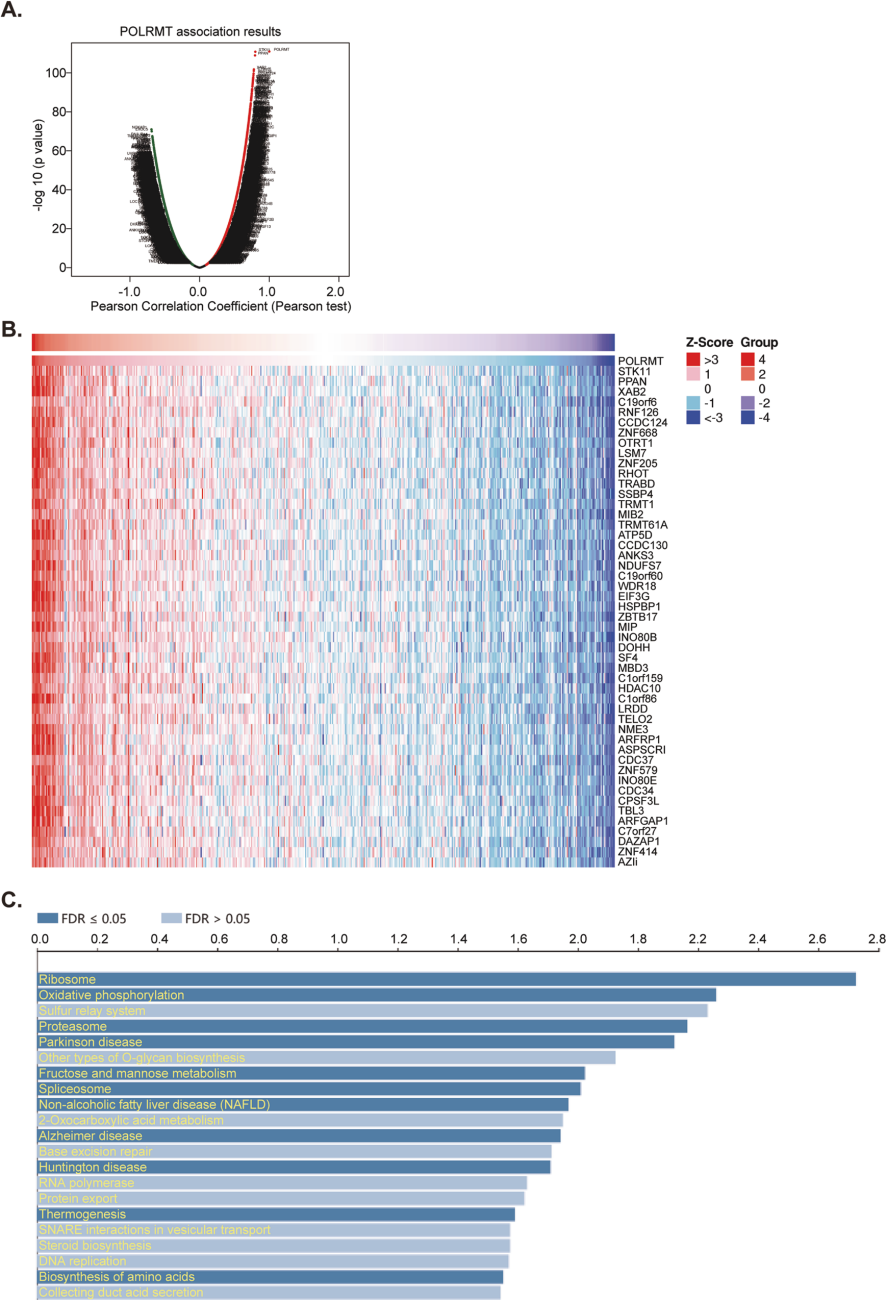
POLRMT co-expressing genes (CEGs) and enriched pathways in TCGA prostate cancer cohort. The Cancer Genome Atlas prostate cancer cohort (TCGA PRAD) and LinkedOmics functional studies demonstrated *POLRMT* co-expressing genes (CEGs) in prostate cancer tissues (**A**). The top 50 positively-correlating CEGs with *POLRMT* were presented (**B**). KEGG analyses showed the top twenty (20) enriched pathways of *POLRMT* positively-correlating *CEGs* (**C**).

To explore the possible functional role of PORLMT in prostate cancer, the mRNA sequencing data from 501 prostate cancer tissues in TCGA prostate cancer cohort was analyzed using the LinkedOmics functional module. The volcano plots showed the co-expressed genes (CEGs) with *POLRMT* (Fig. 3A). Of which, CEGs positively-correlating with *POLRMT* were in red dots and CEGs negatively-correlating with *POLRMT* were in green dots (false discovery rate [FDR] <0.01) (Fig. 3A). The top fifty (50) CEGs positively-correlating with POLRMT in the prostate cancer tissues were shown in the heat map (Fig. 3B). The significant KEGG (Kyoto encyclopedia of genes and genomes) term annotation by overrepresentation enrichment analysis (ORA) showed the top twenty (20) pathways that were enriched by *POLRMT-*associated CEGs (Fig. 3C). These cascades primarily participated in regulating mitochondrial functions, biosynthesis, and metabolism (Fig. 3C), including “Oxidative phosphorylation”, “RNA polymerase”, “DNA replication” and “Biosynthesis of amino acids”, among others (Fig. 3C). These results supported a potential role of POLRMT in regulating mitochondrial functions in prostate cancer.

### POLRMT depletion impairs mitochondrial functions in prostate cancer cells

**Fig. 4:**
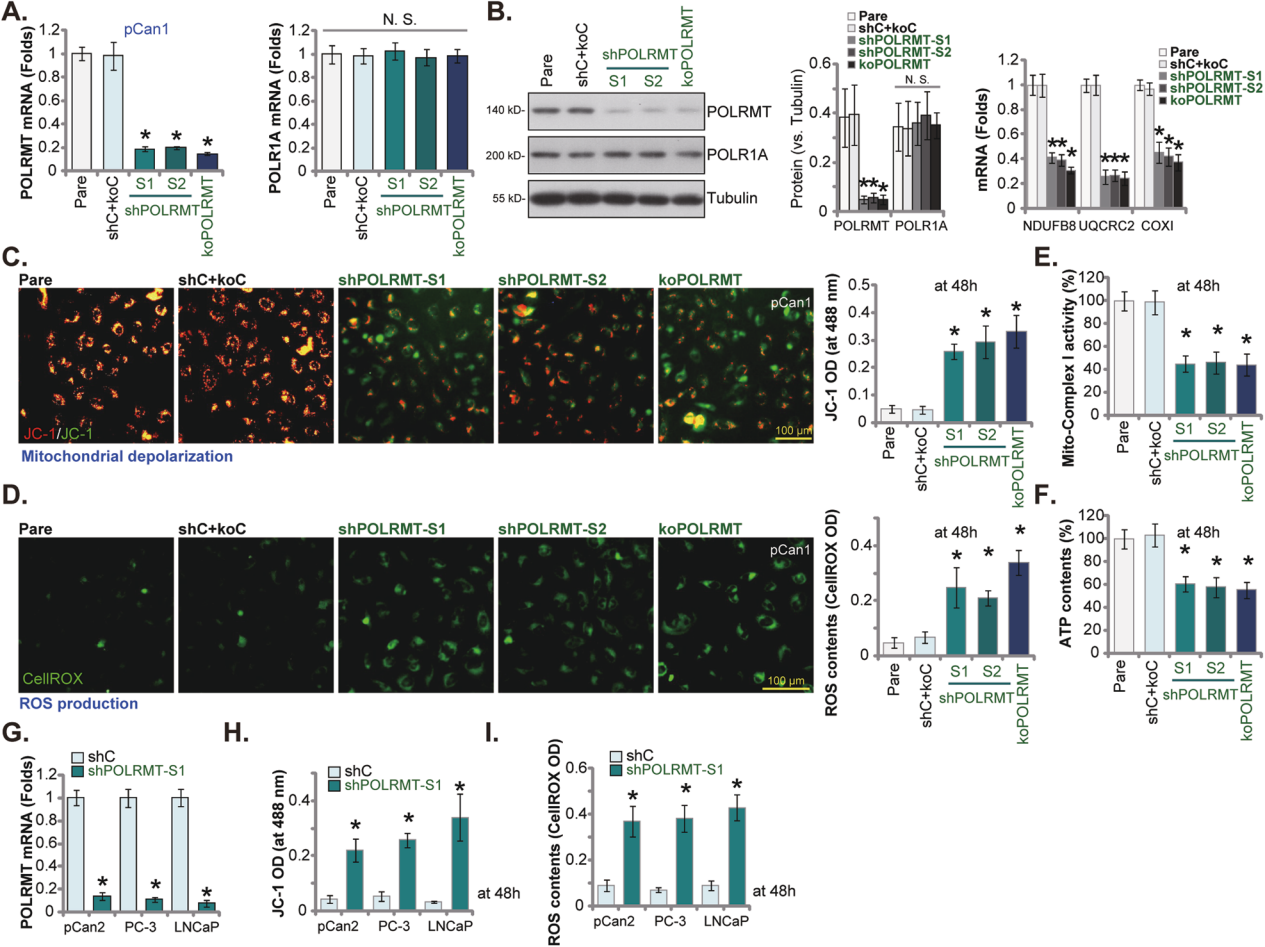
POLRMT depletion impairs mitochondrial functions in prostate cancer cells. The pCan1 primary prostate cancer cells with the described lentiviral POLRMT shRNA (“shPOLRMT-S1 and shPOLRMT-S2”), the lentiviral CRISPR/Cas9-POLRMT-KO construct (“koPOLRMT”), or the lentiviral scramble control shRNA plus CRISPR/Cas9 control construct (“shC+koC”), were established, and expression of listed mRNAs and proteins was tested (**A**, **B**). Cells were further cultivated for 48h, mitochondrial depolarization (by measuring JC-1 monomers) (**C**) and ROS production (CellROX intensity) (**D**) as well as the mitochondria respiratory china complex I activity (**E**) and cellular ATP contents (**F**) were measured. The primary prostate cancer cells (“pCan2”) and the immortalized lines (PC-3/LNCaP), with the lentiviral POLRMT shRNA (“shPOLRMT-S1”) or the lentiviral scramble control shRNA (“shC”), were established and expression of POLRMT mRNA was shown) (**G**); Cells were further cultivated for 48 h, JC-1 green monomer intensity (**H**) and ROS production (by measuring CellROX green fluorescence intensity, (**I**) were tested. Data were presented as mean ± standard deviation (SD, *n* = 5). “Pare” stands for the parental control cells. *P < 0.05 vs. “shC+koC”/“shC” cells. “N. S.” stands for the non-statistical difference (*P* > 0.05). The experiments were repeated five times with similar results obtained. Scale bar = 100 μm.

We thus explored whether POLRMT depletion using genetic means affected mitochondrial functions in prostate cancer cells. First two different lentiviral shRNAs, shPOLRMT-S1 and shPOLRMT-S2 (from Dr. Zhou [20]), were individually transduced to pCan1 primary prostate cancer cells. Stable cells were then formed after selection by puromycin. Alternatively, a lentiviral CRISPR/Cas9-POLRMT-KO construct (from Dr. Li [22]) was employed to knockout POLRMT in pCan1 cells (“koPOLRMT”). As compared to the control pCan1 cells with lentiviral scramble control shRNA and CRISPR/Cas9 control construct (“shC+koC”), *POLRMT* mRNA (Fig. 4A) and protein (Fig. 4B) expression was substantially decreased in shPOLRMT-S1/S2 pCan1 cells and koPOLRMT pCan1 cells. Expression of *POLR1A* mRNA and protein (the control) was unchanged (Fig. 4A, B). The number of POLRMT-dependent mitochondrial transcripts, including *NDUFB8*, *UQCRC2*, and *COXI* [13, 18, 20–22, 30], was decreased as well after POLRMT silencing/KO in pCan1 cells (Fig. 4B).

Importantly, POLRMT shRNA or KO impaired mitochondrial functions and led to mitochondrial depolarization (Fig. 4C). It was evidenced by JC-1 transition from red fluorescence polymers to green fluorescence monomers (Fig. 4C). In Fig. 4D, increased CellROX fluorescence staining supported ROS production and oxidative stress in POLRMT-silenced/-KO pCan1 cells. In addition, the mitochondria respiratory china complex I activity (Fig. 4E) and cellular ATP contents (Fig. 4F) were both decreased in POLRMT-silenced/-KO pCan1 cells. The control genetic treatments, shC+koC, failed to significantly alter POLRMT and POLR1A expression (Fig. 4A, B) and mitochondrial functions (Fig. 4C–F) in pCan1 cells.

In the other patient-derived prostate cancer cells (“pCan2”) and immortalized lines (PC-3/LNCaP), the lentiviral shPOLRMT-S1 was stably transduced to silence *POLRMT* (Fig. 4G). POLRMT silencing led to significant mitochondrial depolarization and ROS production in the primary/established prostate cancer cells, evidenced by increasing in JC-1 green fluorescence monomer intensity (Fig. 4H) and CellROX red fluorescence intensity increasing (Fig. 4I), respectively. Together, POLRMT depletion impaired mitochondrial functions in prostate cancer cells.

### POLRMT depletion impedes prostate cancer cell survival, proliferation and migration

**Fig. 5:**
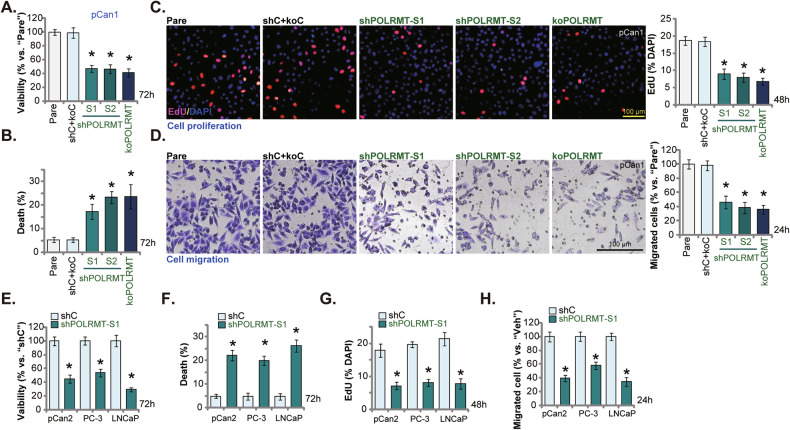
POLRMT depletion impedes prostate cancer cell viability, proliferation and migration. The primary pCan1 cells with the described genetic modification on POLRMT or the control genetic treatment (“shC+koC”) were established and cells were cultivated for the described time. Cell viability (CCK-8 assay) (**A**), death (by measuring Trypan blue-positive cells’ ratio) (**B**), proliferation (nuclear EdU incorporation) (**C**), and migration (“Transwell” assays) (**D**) were tested. The pCan2 primary prostate cancer cells and the immortalized lines (PC-3/LNCaP), with the lentiviral POLRMT shRNA (“shPOLRMT-S1”) or the lentiviral scramble control shRNA (“shC”), were established and cultivated for described time periods, cell viability (**E**), death (**F**), proliferation (**G**) and migration (**H**) were tested similarly, with results quantified. Data were presented as mean ± standard deviation (SD, *n* = 5). “Pare” stands for the parental control cells. *P < 0.05 vs. “shC+koC”/“shC” cells. The experiments were repeated five times with similar results obtained. Scale bar = 100 μm.

We next tested whether POLRMT depletion altered prostate cancer cell behaviors. The cell viability was measured by CCK-8 assay and results showed that POLRMT silencing (by shPOLRMT-S1/S2, see Fig. 4) or KO (by CRISPR/Cas9 method, see Fig. 4) decreased viability (CCK-8 OD) in pCan1 primary cancer cells (Fig. 5A). By increasing Trypan blue staining, POLRMT depletion by the genetic means resulted in pCan1 cell death (Fig. 5B). The EdU-positive nuclei ratio was substantially decreased after POLRMT silencing/KO in pCan1 cells (Fig. 5C), suggesting that genetic POLRMT depletion inhibited prostate cancer cell proliferation (Fig. 5C). In addition, “Transwell” assay results demonstrated that pCan1 cell in vitro migration (Fig. 5D) was potently inhibited after PLORMT silencing/KO. The control shC+koC genetic treatments had no significant effect on the pCan1 cell behaviors (Fig. 5A–D). In pCan2 cells and immortalized lines (PC-3/LNCaP), POLRMT silencing by shPOLRMT-S1 (see Fig. 4) similarly induced viability reduction (Fig. 5E) and cell death (Fig. 5F). Moreover, proliferation (EdU-nuclei ratio, Fig. 5G) and migration (Fig. 5H) were significantly inhibited after POLRMT silencing in the prostate cancer cells.

### POLRMT depletion induces prostate cancer cell apoptosis

**Fig. 6:**
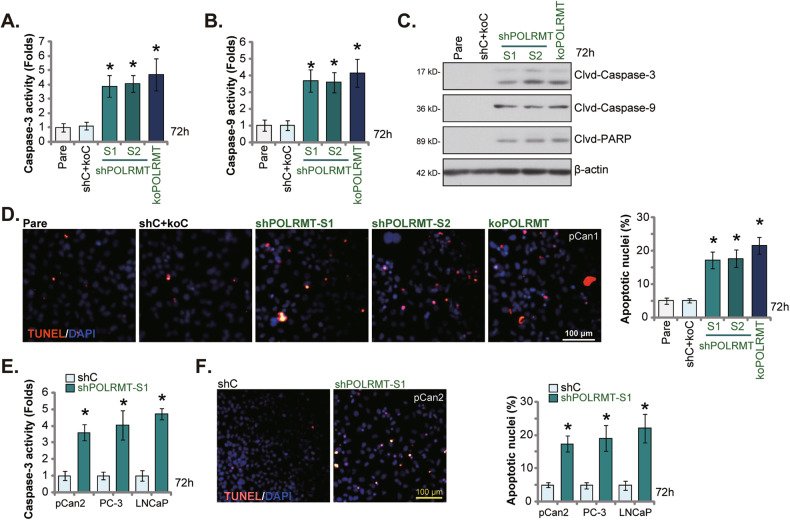
POLRMT depletion induces prostate cancer cell apoptosis. The primary pCan1 cells with the described genetic modification on POLRMT or the control genetic treatment (“shC + koC”) were established and cells were cultivated for 72h. The Caspase-3 activity (**A**), the Caspase-9 activity (**B**), and the expression of listed apoptosis-associated proteins (**C**) were tested; Cell apoptosis was measured by nuclear TUNEL staining assay (**D**). The primary prostate cancer cells (“pCan2”) and the immortalized cell lines (PC-3/LNCaP), with the lentiviral POLRMT shRNA (“shPOLRMT-S1”) or the lentiviral scramble control shRNA (“shC”), were established and cultivated for 72h; The Caspase-3 activity (**E**) and cell apoptosis (**F**) were tested similarly. Data were presented as mean ± standard deviation (SD, *n* = 5). “Pare” stands for the parental control cells. *P < 0.05 vs. “shC+koC”/“shC” cells. The experiments were repeated five times with similar results obtained. Scale bar = 100 μm.

The potential effect of genetic POLRMT depletion on cell apoptosis was examined next. In pCan1 cells, POLRMT silencing (by shPOLRMT-S1/S2) or KO (by CRISPR/Cas9 method) significantly increased Caspase-3 (Fig. 6A) and Caspase-9 (Fig. 6B) activities. Moreover, increased cleavages of Caspase-3, Caspase-9 and poly (ADP-ribose) polymerase 1 (PARP) were detected in POLRMT-silenced/-KO pCan1 cells (Fig. 6C). To support apoptosis activation, we showed that TUNEL-positive nuclei ratio was robustly increased in POLRMT-depleted pCan1 cells (Fig. 6D). The control shC+koC genetic treatments, expectably, failed to provoke caspase-apoptosis activation in pCan1 primary cells (Fig. 6A–D). In pCan2 primary cells and immortalized lines (PC-3/LNCaP), shPOLRMT-S1-induced silencing of POLRMT (see Figs. 4, 5) similarly increased Caspase-3 activity (Fig. 6E). shPOLRMT-S1 treatment also increased TUNEL-positive nuclei ratio (Fig. 6F) in the primary and immortalized cancer cells. Thus, POLRMT depletion induced apoptosis activation in prostate cancer cells.

### POLRMT overexpression induces pro-cancerous activity in primary prostate cancer cells

**Fig. 7:**
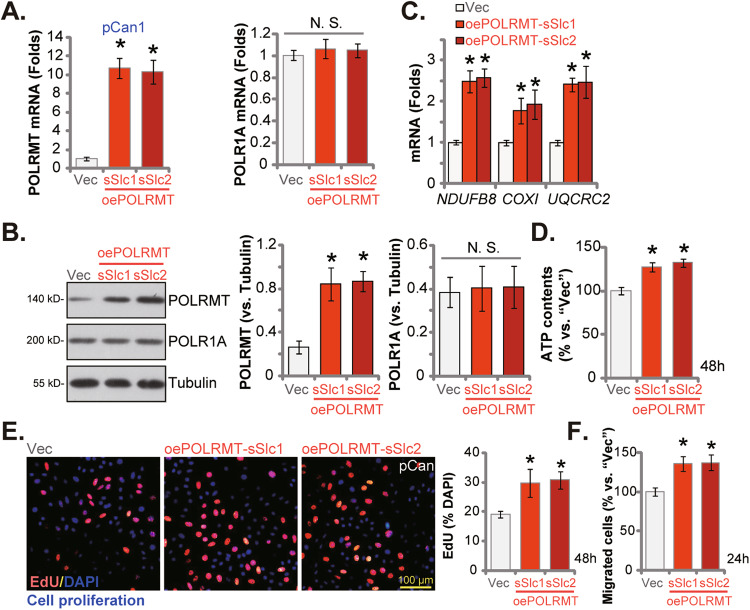
POLRMT overexpression induces pro-cancerous activity in primary prostate cancer cells. The primary pCan1 cells with the lentiviral POLRMT-expressing construct (“oePOLRMT-sSlc1” and “oePOLRMT-sSlc2”, two stable selections) or the empty vector (“Vec”) were established and expression of listed mRNAs and proteins was shown (**A**–**C**); Cells were further cultivated for indicated time periods and cellular ATP contents were measured (**D**); Cell proliferation (nuclear EdU incorporation) (**E**) and migration (“Transwell” assays) (**F**) were tested as well. Data were presented as mean ± standard deviation (SD, *n* = 5). *P < 0.05 vs. “Vec” cells. “N. S.” stands for the non-statistical difference (*P* > 0.05). The experiments were repeated five times with similar results obtained. Scale bar = 100 μm.

The above results showed that POLRMT silencing or KO led to robust anti-cancer activity in different prostate cancer cells. We thus hypothesized that ectopic overexpression of POLRMT could possibly induce the opposite effect. To test this hypothesis, the lentivirus encoding the POLRMT-expressing construct (“oePOLRMT”) was added to the pCan1 primary cells, and puromycin was added to select two stable selections: “oePOLRMT-sSlc1” and “oePOLRMT-sSlc2”. Expression of *POLRMT* mRNA (Fig. 7A) and protein (Fig. 7B) was remarkably increased in oePOLRMT-expressing pCan1 cells. Whereas *POLR1A* mRNA and protein expression was unaltered (Fig. 7A, B). Levels of POLRMT-dependent mitochondrial transcripts, *NDUFB8*, *UQCRC2*, and *COXI*, were increased in pCan1 cells with PLORMT overexpression (Fig. 7C). Moreover, the cellular ATP contents were elevated (Fig. 7D). Ectopic overexpression of POLRMT promoted pCan1 cell proliferation and increased nuclear EdU incorporation (Fig. 7E). Moreover, pCan1 cell in vitro migration was accelerated with POLRMT overexpression (Fig. 7F). These results further supported the pro-cancerous role of POLRMT in prostate cancer cells.

### POLRMT silencing impedes prostate cancer xenograft growth in nude mice

**Fig. 8:**
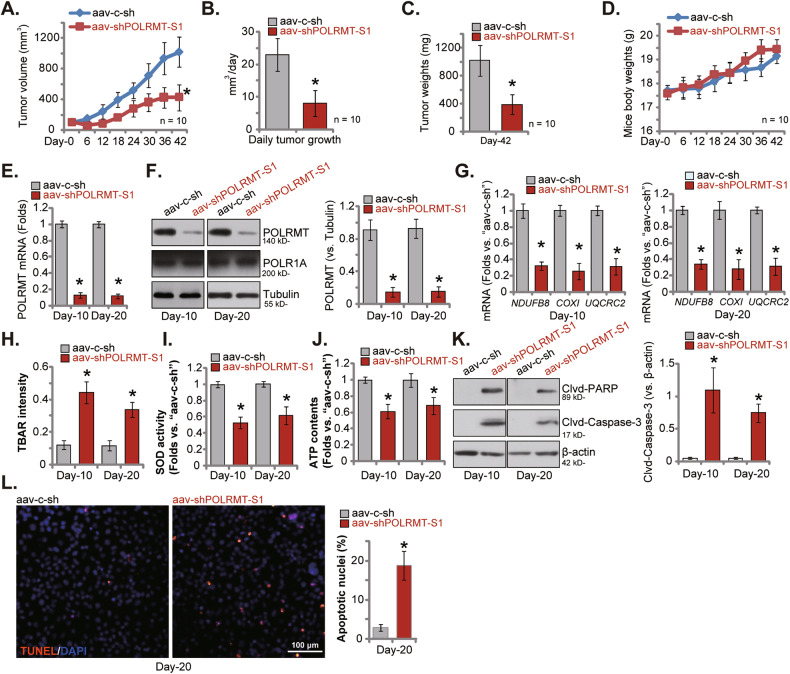
POLRMT silencing impedes prostate cancer xenograft growth in nude mice. The nude mice bearing pCan1 xenografts were subject to intratumoral injection of POLRMT shRNA-expressing AAV (“aav-shPOLRMT-S1”) or control shRNA-expressing AAV (“aav-c-sh”). The pCan1 xenograft volumes (**A**) and nude mice body weights (**D**) were recorded every six days; The daily pCan1 xenograft growth, in mm^3^ per day, was estimated (**B**). At Day-42, pCan1 xenografts were all isolated carefully and weighted individually (**C**). At Day-10 and Day-20, one pCan1 xenograft of each group was isolated, tissue lysates were obtained and expression of listed mRNAs and proteins in the lysates was shown (**E**–**G** and **K**); The TBAR intensity (**H**), SOD activity (**I**) and ATP contents (**J**) in the tissue lysates were examined as well. Alternatively, the pCan1 xenograft tissue slides were tested via immunofluorescence staining of TUNEL/DAPI (**L**). Data were presented as mean ± standard deviation (SD). In (**A**–**D**), *n* = 10 stands for ten mice per group. For (**E**–**L**), five random tissues in each xenograft were tested. *P < 0.05 versus “aav-c-sh” group. Scale bar = 100 μm.

To support the role of POLRMT in the growth of prostate cancer cells in vivo, the subcutaneous xenograft model was established. Specifically, pCan1 primary cells (at six million cells in each mouse) were subcutaneously (*s.c*.) injected into the flanks of the nude mice. Within three weeks, pCan1 xenografts were formed and each xenograft was ~100 mm^3^ in total volume (labeled as “Day-0”). POLRMT shRNA-expressing AAV (“aav-shPOLRMT-S1”, from Dr. Zhou [20]) or control shRNA-expressing AAV (“aav-c-sh”, also from Dr. Zhou [20]) were then injected to the xenografts. The virus was injected twice (at “Day-0” and “Day-2”). As shown, aav-shPOLRMT-S1 injection remarkably inhibited pCan1 xenograft growth in nude mice (Fig. 8A). The volumes of shPOLRMT-S1 xenografts were much lower than those of aav-c-sh xenografts (Fig. 8A). Results from the estimated daily pCan1 xenograft growth, in mm^3^ per day, further showed that pCan1 xenograft growth was remarkably suppressed after aav-shPOLRMT-S1 injection (Fig. 8B). Forty-two days after initial virus injection (“Day-42”), pCan1 xenografts were isolated and weighted. Again, aav-shPOLRMT-S1-treated pCan1 xenografts were significantly lighter than the aav-c-sh-administrated ones (Fig. 8C). The animal body weights were however indifferent between aav-shPOLRMT-S1-treated mice and aav-c-sh-administrated ones (Fig. 8D).

At “Day-10” (ten days after initial virus injection) and “Day-20” (twenty days after initial virus injection), one pCan1 xenograft of each group was isolated through surgery, and tissues of the four xenografts were analyzed. As shown, *POLRMT* mRNA (Fig. 8E) and protein (Fig. 8F) expression were substantially decreased in aav-shPOLRMT-S1-treated xenograft tissues. mRNA expression of POLRMT-dependent mitochondrial genes (*NDUFB8*, *UQCRC2*, and *COXI*), was downregulated after POLRMT silencing (Fig. 8G). The TBAR activity was enhanced in POLRMT-silenced pCan1 xenograft tissues (Fig. 8H) and the SOD activity was decreased (Fig. 8I). Moreover, ATP contents were decreased in xenograft tissues with aav-shPOLRMT-S1 injection (Fig. 8J). Injection of POLRMT shRNA AAV resulted in apoptosis in pCan1 xenograft tissues. Cleavages of Caspase-3 and PARP were strengthened in aav-shPOLRMT-S1-treated xenografts (Fig. 8K). In addition, in POLRMT-silenced xenograft tissue slides, the TUNEL-positively stained nuclei were significantly increased (Fig. 8L). These signaling results supported that POLRMT silencing caused mitochondrial dysfunction, oxidative injury, ATP depletion, and apoptosis in pCan1 xenograft tissues. The in vivo findings were thus consistent with in vitro findings.

### IMT1, the first-in-class POLRMT inhibitor, inhibits prostate cancer cell growth in vitro and in vivo

**Fig. 9:**
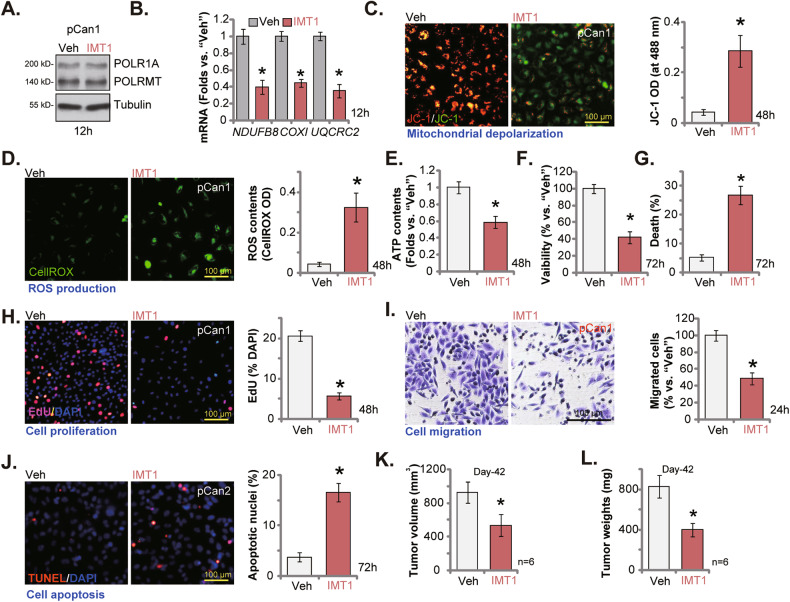
IMT1, the first-in-class POLRMT inhibitor, inhibits prostate cancer cell growth in vitro and in vivo. The primary pCan1 cells were treated with IMT1 (0.5 μM) or vehicle control (“Veh”) and cultured for the described hours, expression of listed proteins and mRNAs was tested (**A**, **B**); Mitochondrial depolarization (by measuring JC-1 monomers, (**C**)), ROS production (CellROX intensity, (**D**)) and ATP contents (**E**) were measured. Cell viability (CCK-8 assays, (**F**)), death (by measuring Trypan blue-positive cells’ ratio, (**G**)), proliferation (nuclear EdU incorporation, (**H**)), migration (“Transwell” assays, (**I**)) and apoptosis (TUNEL nuclear staining, (**J**)) were tested as well. The nude mice bearing pCan1 xenografts were orally-administrated with IMT1 (50 mg/kg body, every 48 h) or the vehicle control (“Veh”). At Day-42, pCan1 xenografts were all isolated carefully, and tumor volumes (**K**) and tumor weights (**L**) were measured. Data were presented as mean ± standard deviation (SD). *P < 0.05 vs. “Veh” group. The in vitro experiments were repeated five times with similar results obtained. For animal xenograft studies, *n* = 6 stands for six mice per group. Scale bar = 100 μm.

At last, we tested the potential effect of IMT1, the first-in-class POLRMT inhibitor [22, 30], in prostate cancer cells. Referring to early studies, IMT1 was utilized at 0.5μM in vitro [22, 30]. It did not alter POLRMT and POLR1A protein expression in pCan1 cells (Fig. 9A). POLRMT-dependent transcripts (*NDUFB8*, *UQCRC2* and *COXI*) were however downregulated in IMT1-treated pCan1 cells (Fig. 9B). Treatment with the POLRMT inhibitor resulted in mitochondrial depolarization (JC-1 monomers accumulation, Fig. 9C) and ROS production (Fig. 9D). ATP contents were decreased as well (Fig. 9E). Moreover, IMT1 caused significant viability reduction (Fig. 9F) and cell death (Fig. 9G) in pCan1 cells. The POLRMT inhibitor also suppressed pCan1 cell proliferation (EdU nuclear incorporation, Fig. 9H) and migration (Fig. 9I), while provoking apoptosis (Fig. 9J). These results clearly showed that POLRMT inhibition by IMT1 impaired mitochondrial functions and inhibited primary prostate cancer cell growth.

To explore the potential effect of IMT1 on prostate cancer cell growth in vivo, pCan1 xenograft (close to 100 mm^3^ in volume)-bearing mice were administrated with IMT1 at (50 mg/kg body weights), every 48 h for five rounds [22]. Forty two days after initial IMT1 administration (“Day-42”), xenografts were separated and analyzed. As shown, IMT1-treated pCan1 xenografts were significantly smaller (Fig. 9K) and lighter (Fig. 9L) than vehicle control (“Veh”)-treated xenografts. No significant difference in the mice body weights was detected and no apparent toxicities were noticed. Thus, IMT1 administration hindered pCan1 xenograft growth in nude mice.

## Discussion

Mitochondria are key determinants for oncogenesis and progression of prostate cancer [10–12]. The mitochondrial transcriptional machinery and subsequent protein translation are both elevated, which are associated with poor prognosis of patients with different cancers, including acute myeloid leukemia (AML), breast cancer, lung cancer, and several others [18, 20, 31–35]. A number of different cancer cells are dependent on oxidative phosphorylation (OXPHOS) for the supply of ATP and biosynthetic intermediates [36, 37]. Unlike other cancerous cells that mainly utilize inefficient aerobic glycolysis to provide energy, the tumorigenesis of prostate cancer is associated with the transition from inefficient metabolism to efficient mitochondrial metabolism [38].

Dysregulation of mitochondria plays an important role in oncogenesis and progression of prostate cancer [10–12]. Ippolito et al. found that the mitochondrial mass and activity were both augmented in prostate cancer cells possibly due to lactate uptake from cancer-associated fibroblasts [39]. Zhao et al. reported that the mitochondrial protein PPFIA4 (protein tyrosine phosphatase receptor type F polypeptide interacting protein alpha 4) was increased in prostate cancer and promoted prostate cancer cell growth via enhancing mitochondrial metabolism [40]. Kumar et al. reported that the mitochondrial chaperonin heat shock protein 60 (HSP60) interaction with the mitochondrial protease caseinolytic protease P (ClpP) was vital for maintaining mitochondrial functions and prostate cancer cell survival [41]. Contrarily, disruption of HSP60-ClpP interaction caused metabolic stress and impeded prostate cancer cell growth and progression [41].

The results of the present study proposed an important role of the mitochondrial protein POLRMT in prostate cancer. TCGA results show that *POLRMT* transcripts are increased in prostate cancer tissues and *POLRMT* overexpression correlated with poor overall survival of the patients. In addition, *POLRMT* mRNA and protein levels are significantly elevated in local prostate cancer tissues and in different primary/immortalized cancer cells. Genetic depletion of *POLRMT*, using viral shRNA or CRISPR/Cas9 method, resulted in substantial inhibition on prostate cancer cell viability, proliferation, and migration, while provoking apoptosis. Contrarily, prostate cancer cell proliferation and migration were strengthened after ectopic POLRMT overexpression. In vivo, intratumoral injection of POLRMT shRNA-expressing AAV impeded prostate cancer xenograft growth in nude mice. Importantly, IMT1, the first-in-class POLRMT inhibitor, inhibited prostate cancer cell growth in vitro and in vivo. Therefore, overexpressed POLRMT is important for prostate cancer cell growth, representing as a novel diagnostic and therapeutic target.

A group of recent findings have shown that POLRMT silencing/inhibition impaired mitochondrial functions in cancerous cells. The mtDNA contents, mitochondrial transcripts, and respiratory chain complex subunits were all downregulated in POLRMT-depleted NSCLC cells [20] and osteosarcoma cells [19]. Wang et al., discovered that POLRMT silencing resulted in depolarization of mitochondria, oxidative injury, and ATP depletion in skin squamous cell carcinoma (SCC) cells in vitro and in vivo [21]. A very recent study discovered that POLRMT inhibition by IMT1 resulted in mtDNA transcription inhibition, mitochondrial oxidative injury, energy stress, and ATP loss in endometrial carcinoma cells [22].

Here we found that POLRMT is key in maintaining mitochondrial functions in prostate cancer cells. In primary prostate cancer cells, POLRMT shRNA or KO led to mitochondrial depolarization, ROS production, mitochondria complex I inhibition, and ATP reduction. Contrarily, ATP contents were augmented following ectopic POLRMT overexpression. Inhibition of POLRMT by IMT1 also impaired mitochondrial functions in primary prostate cancer cells, resulting in mitochondrial depolarization, ROS production, and ATP reduction. mRNA expression of key mitochondrial genes, including *NDUFB8*, *UQCRC2*, and *COXI*, was decreased with POLRMT silencing or inhibition, but increased with POLRMT overexpression in prostate cancer cells. Importantly, in POLRMT-silenced prostate cancer xenograft tissues, *NDUFB8*, *UQCRC2*, and *COXI* downregulation as well as oxidative stress, lipid peroxidation, and ATP reduction were detected as well. Thus, POLRMT depletion impaired mitochondrial functions and impeded prostate cancer cell growth in vitro and in vivo.

## Conclusion

Therefore, overexpressed POLRMT is an important mitochondrial protein for prostate cancer cell growth, representing as a novel and promising diagnostic/therapeutic target.

### Supplementary information

checklist FORM

Author contribution form

Original Data File

## Data Availability

All data are available upon request.
