# Supplementary material for: Identification of the mitochondrial protein POLRMT as a potential therapeutic target of prostate cancer
Source: Cell Death Dis. 2023 Oct 10;14(10):665. doi: 10.1038/s41419-023-06203-2 (PMC10564732; doi:10.1038/s41419-023-06203-2)
Supplement: Supplementary file 2 — Author contribution form [file 41419_2023_6203_MOESM2_ESM.pdf]

DECLARATION OF CONTRIBUTIONS TO ARTICLE

ADMC

Manuscript Number:	Journal Name:	
CDDIS-23-1969	*Cell Death & Disease*	(the 'Journal')

Proposed Title of the Contribution:

Identification of the mitochondrial protein POLRMT as a potential therapeutic target of prostate cancer (the 'Contribution)

Author(s):

Xiaojun Li, Linya Yao, Tao Wang, Xiaolei Gu, Yufan Wu and Ting Jiang (the 'Authors')

For all *CDDis* articles, each person named as an author in the published version must be able to show he or she has contributed substantially to the article.

Authorship credit should be based on 1) substantial contributions to conception and design, acquisition of data, or analysis and interpretation of data; 2) drafting the article or revising it critically for important intellectual content; and 3) final approval of the version to be published. Authors should meet conditions 1, 2 and 3.

Any person who cannot be shown to have made a substantial contribution to the article cannot be listed as an author in the final version. The name of any person who is deemed to have made a minor contribution can, however, appear in the Acknowledgments section of the article.

Please complete the table below to indicate the contributions of all named authors to the manuscript.

Author Full Name:	Specification of Contribution to the Manuscript:
Xiaojun Li	Functional assays in prostate cancer cells, establishing animal xenograft models, testing signalings in cells and tissues and performing cellular functional assays, drafting the article and revising it, with final approval of the version submitted to the journal.
Linya Yao	Functional assays in prostate cancer cells, establishing animal xenograft models, testing signalings in cells and tissues and performing cellular functional assays, drafting the article and revising it, with final approval of the version submitted to the journal.
Tao Wang	Functional assays in prostate cancer cells, establishing animal xenograft models, testing signalings in cells and tissues and performing cellular functional assays, drafting the article and revising it, with final approval of the version submitted to the journal.
Xiaolei Gu	Functional assays in prostate cancer cells, establishing animal xenograft models, testing signalings in cells and tissues and performing cellular functional assays, drafting the article and revising it, with final approval of the version submitted to the journal.
Yufan Wu	Functional assays in prostate cancer cells, establishing animal xenograft models, testing signalings in cells and tissues and performing cellular functional assays, drafting the article and revising it, with final approval of the version submitted to the journal.
Ting Jiang	Study conception and design, and data analysis, Figure organization, involved in drafting the article and revising it critically for important intellectual content, and with final approval of the version submitted to the journal. .

Please complete the table below to indicate the contributions of all named authors to the figures.

Figure 1:

Xiaojun Li, Linya Yao, Tao Wang, Xiaolei Gu, Yufan Wu and Ting Jiang

Figure 2:

Xiaojun Li, Linya Yao, Tao Wang, Xiaolei Gu, Yufan Wu and Ting Jiang

Figure 3:

Xiaojun Li, Linya Yao, Tao Wang, Xiaolei Gu, Yufan Wu and Ting Jiang

Figure 4:

Xiaojun Li, Linya Yao, Tao Wang, Xiaolei Gu, Yufan Wu and Ting Jiang

Figure 5:

Xiaojun Li, Linya Yao, Tao Wang, Xiaolei Gu, Yufan Wu and Ting Jiang

Figure 6:

Xiaojun Li, Linya Yao, Tao Wang, Xiaolei Gu, Yufan Wu and Ting Jiang

Figures 7-9

Xiaojun Li, Linya Yao, Tao Wang, Xiaolei Gu, Yufan Wu and Ting Jiang

Signed for and on behalf of the Author(s):	Print Name:	Date:
	Ting Jiang	05-21-2023
