# Supplementary material for: Identification of the mitochondrial protein POLRMT as a potential therapeutic target of prostate cancer
Source: Cell Death Dis. 2023 Oct 10;14(10):665. doi: 10.1038/s41419-023-06203-2 (PMC10564732; doi:10.1038/s41419-023-06203-2)
Supplement: Supplementary file 3 — Original Data File [file 41419_2023_6203_MOESM3_ESM.pdf]

Figure S1.

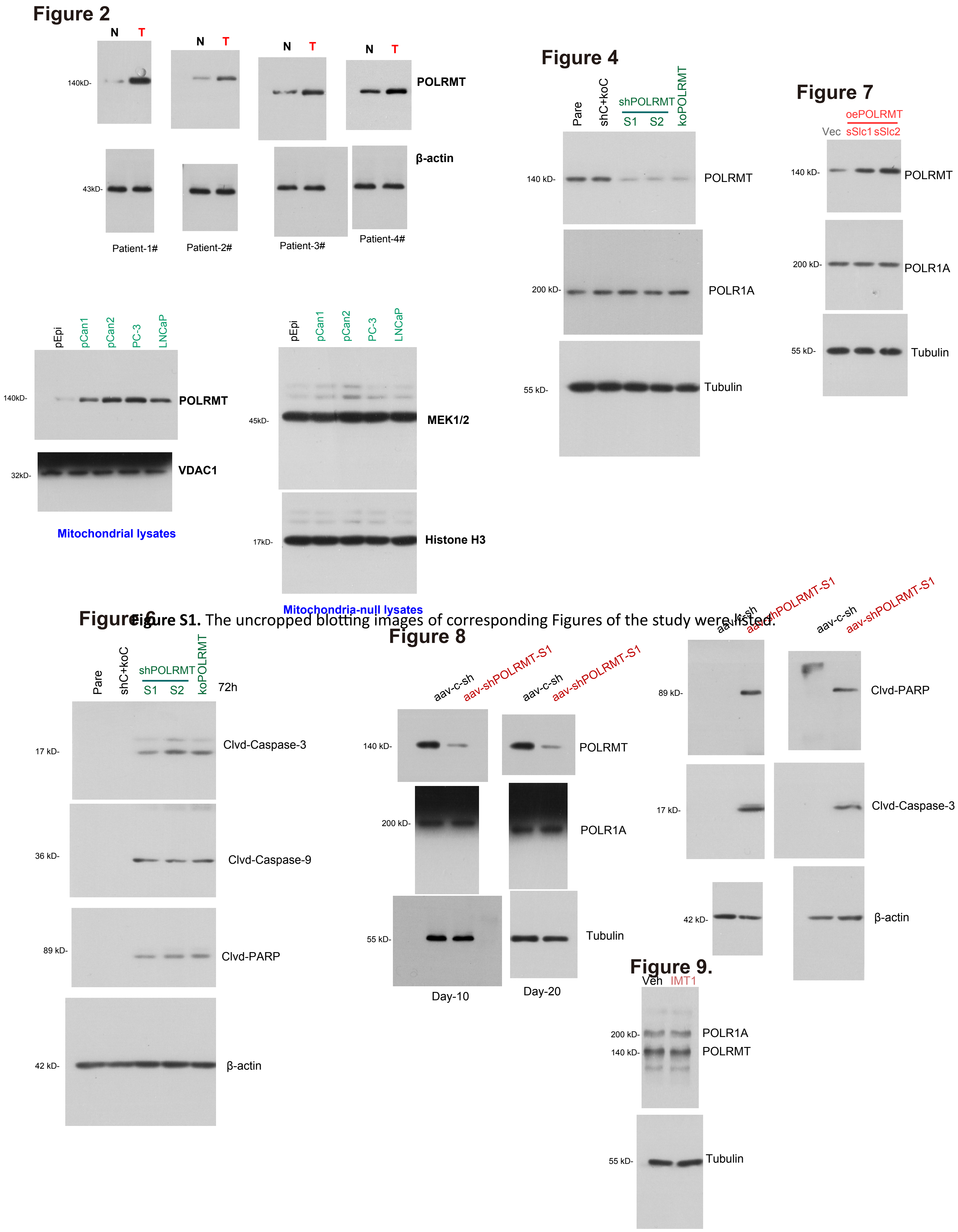

Figure S1. The uncropped blotting images of corresponding Figures of the study were listed.
